# An Outline for the Treatment of Eczema

**Published:** 1912-12

**Authors:** W. W. Quinton

**Affiliations:** Buffalo, N. Y.


### An Outline for the Treatment of Eczema

By W. W. QUINTON, M.D.

Buffalo, N. Y.

ONE might think that the last word had long since been said so far as the treatment of eczema is concerned. We are, however, daily reminded of the fact that this is not so, by the number of cases of this disease which go floating about from doctor to doctor, from general practitioner to specialist; their restless wanderings bearing silent testimony to the efficacy of their treatment.

The importance of the subject should be evident to even the most casual thinker if he will reflect that twenty-seven per cent. of all the diseases of the skin are classified under the heading of "eczema."

If I were called upon to state what is the first and most important step in the treatment of any cutaneous affection, I should unhesitatingly say, a correct diagnosis—and this certainly applies to the consideration of eczema. For example: One must expect a syphilitic eruption to prove more or less resistant if the patient is treated for eczema. A toxic dermatitis may assume the appearance of an eczema at some time during its course, but your eczema treatment will fail, unless you arrive at a correct diagnosis and remove the cause. Do not promise to cure your patient of eczema of the face when, in reality, he has lupus erythematosus. This advice may sound superfluous but these same mistakes—and many similar ones—have been made in the past and will probably be made again in the future.

It is a good working principle, in dealing with this condition, to start the treatment by searching for a cause. You will not always find it, but nevertheless, the effort is worth while. Not all observers believe in combined local and general treatment, but it has always seemed to me that ordinary common-sense would indicate a method of treatment, both medicinal and hygienic, which should have for its object the return of the patient to a condition of the best possible state of bodily health. Having attained this, one may certainly expect to limit, to a degree, the eczematous attacks. The close observance of fixed hygienic rules must be insisted upon. The patient's diet must receive the most careful attention and all harmful food articles should be interdicted. Certainly it would occur to any of us, to relieve constipation at once, and to examine most thoroughly into the condition of the kidneys.

Let us now turn to a consideration of the local treatment. Here, many of us fail, for the reason that we do not accord to the disease the proper amount of respect. If we are not very familiar with its treatment, we reach for a medical book and try one of the many formulae which we find therein. Or, on the other hand, it may be that we have a stock remedy for all such cases regardless of their characteristics and individual peculiarities. Do not flatter yourself that you can give the patient this or that valuable prescription, with directions to anoint himself so many times a day with it, and blithely expect him to return to your office in a few days, cured and ready to fall on your neck in his gratitude. It will not happen this way. If you expect to get any results which will redound to your credit, you must make up your mind to devote a considerable amount of time to your patient. Every day will not be too often to see him and to give him a careful examination. The sharper and more observant your eyes, and the greater your experience with such troubles, the greater will be your success.

The initial treatment of an acute eczema should be the very mildest that we can possibly devise. Never can one make a greater mistake than to use an irritating or stimulating preparation at this stage. The more soothing the preparation, be it ointment or lotion, the quicker the acute symptoms will pass away, and the louder the patient will sing your praises. This antiphlogistic application must be continued until it ceases to benefit. The experience of the physician will tell him when this point has been reached—there is no royal road to this knowledge. The next preparation used must be desicating in its nature to stop the weeping. This is also used until it is seen that it has no longer any effect, when it is replaced in turn by a mild astringent. After a variable time, depending on how quickly the eruption responds to the treatment, this may be changed again and an astringent ointment containing tar, may be substituted.

From this stage onward the strength of the tar is increased gradually until, on some of the more resistant, chronic patches, the pure tar is applied. Avoid the use of tar on the face, however. Here, we are obliged to substitute sulphur.

So much for the management of the ordinary case. The more complex cases will tax the patience of the medical man to the utmost. Variety is the spice of the treatment of eczema, and one's knowledge and ingenuity are constantly challenged by the disease, for prescriptions which will relieve one case, will be found to have little or no effect on another and apparently similar condition. If your application is a little too weak, your case will remain stationary; if a little too strong, expect trouble and you will not be disappointed. The treatment is progessive, and the patient must be graduated from each step to the next higher in the scale. If we ascend too rapidly we are notified of the fact in no uncertain terms, for the eczema will light up with amazing rapidity, to the discomfort of the patient and to our own chagrin. In this event, we have no discretion but to retrace our steps and to begin all over again. One or two such failures will teach us more caution, in this respect, than any number of warnings in a medical article.

The successful treatment of eczema depends on the amount of time and brains the attendant physician is willing to bestow upon it. It sounds simple and it is simple. That the game is well worth the candle, I am convinced. Show me a man whom you have cured of eczema, and I will show you a grateful patient.

